# NEIGHBORHOOD CHARACTERISTICS AND TRAJECTORIES OF DEPRESSIVE SYMPTOMS AND ANXIETY

**DOI:** 10.1093/geroni/igac059.1095

**Published:** 2022-12-20

**Authors:** Weidi Qin

**Affiliations:** University of Michigan, Ann Arbor, Michigan, United States

## Abstract

This study aims to identify the trajectories of mental health among older adults and to examine the association between neighborhood characteristics (i.e. social cohesion and physical disorder) and the identified trajectories. Data came from nine waves of the National Health and Aging Trend Study (N=6,951). Group-based trajectory modeling was used to identify the trajectories of depressive symptoms and anxiety respectively. Multinomial logistic regressions were used to examine the relationship between neighborhood and trajectories. Four trajectories were identified, namely “constantly low” “increasing” “decreasing” and “constantly high”. Results show that higher levels of social cohesion and the absence of physical disorder in the neighborhood demonstrated beneficial and protective effects on older adults’ mental health trajectories. The findings suggest that social cohesion may be an important social capital to cope with negative mental health experiences. On the contrary, negative physical features may be a stressor that adversely affect older adults’ mental health trajectories.

